# Supplementary material for: Generative models struggle with kirigami metamaterials
Source: Sci Rep. 2024 Aug 20;14:19397. doi: 10.1038/s41598-024-70364-z (PMC11339076; doi:10.1038/s41598-024-70364-z)
Supplement: Supplementary file 1 — Supplementary Information. [file 41598_2024_70364_MOESM1_ESM.pdf]

Supplementary material – Generative models struggle with kirigami metamaterials

Gerrit Felsch[1,2] and Viacheslav Slesarenko[1,2,*]

[1]Cluster of Excellence livMatS @ FIT – Freiburg Center for Interactive Materials and Bioinspired Technologies, Freiburg, 79110, Germany
[2]Department of Microsystems Engineering, University of Freiburg, Freiburg, 79110, Germany
[*]viacheslav.slesarenko@livmats.uni-freiburg.de

Neural Network Architectures

The details of the used network architectures are presented in the tables below.

VAE Encoder

Layer	Kernel	Strides	Features	BN	Activation	Padding	Previous
Input I1 6×6	-	-	1	-	-	-	-
Convolution C1	3×3	1×1	8	Yes	LeakyReLU	circular	I1
Convolution C2	5×5	2×2	16	Yes	LeakyReLU	circular	C1
Convolution C3	3×3	1×1	32	Yes	LeakyReLU	circular	C2
Convolution C4	5×5	2×2	64	Yes	LeakyReLU	circular	C3
Flatten F1	-	-	256	No	-	-	C4
Linear L1	-	-	64	No	-	-	F1
Linear L2	-	-	64	No	-	-	F1
Optimizer	Adam lr=1e-4						
Batch size	32						
Epochs	3000						
LeakyReLU slope	0.2						

Table 1. Dimensions and training hyperparameters of the Encoder of the VAE

VAE Decoder + GAN Generator + WGAN Generator

Layer	Kernel	Strides	Features	BN	Activation	Padding	Previous
Input I1	-	-	64	-	-	-	-
Linear L1	-	-	256	No	ReLU	-	I1
Reshape R1 2×2	-	-	64	-	-	-	L1
Transposed Conv T1	3×3	1×1	32	No	ReLU	zeros	R1
Convolution C1	3×3	1×1	32	Yes	ReLU	circular	T1
Transposed ConvT2	3×3	1×1	32	No	ReLU	zeros	C1
Convolution C2	3×3	1×1	1	No	Tanh / None (WGAN)	circular	T2
Optimizer	Adam lr=1e-4 (VAE+WGAN), 1e-5 (GAN)						
Batch size	32						
Epochs	3000						

Table 2. Dimensions and training hyperparameters of the generative parts of the VAE, GAN and WGAN

GAN + WGAN Discriminator

Layer	Kernel	Strides	Features	BN	Activation	Padding	Previous
Input I1 6×6	-	-	1	-	-	-	-
Convolution C1	3×3	1×1	16	Yes	LeakyReLU	circular	I1
Convolution C2	5×5	2×2	32	Yes	LeakyReLU	circular	C1
Convolution C3	3×3	1×1	64	Yes	LeakyReLU	circular	C2
Convolution C4	5×5	2×2	128	Yes	LeakyReLU	circular	C3
Flatten F1	-	-	512	No	-	-	C4
Linear L1	-	-	1	No	Sigmoid / None (WGAN)	-	F1
Optimizer	Adam lr=1e-4 (GAN), 3e-4 (WGAN)						
Batch size	32						
Epochs	3000						
LeakyReLU slope	0.2						

Table 3. Dimensions and training hyperparameters of the discriminators of GAN and WGAN

DDPM

Layer	Kernel	Strides	Features	BN	Activation	Padding	Previous
Input I1 6×6	-	-	1	-	-	-	-
Convolution C1	3×3	1×1	256	Yes	GELU	circular (1)	I1
Convolution C2	3×3	1×1	256	Yes	GELU	circular (1)	C1
Convolution C3	3×3	1×1	256	Yes	GELU	circular (2)	C2
Convolution C4	3×3	1×1	256	Yes	GELU	circular (2)	C3
MaxPool M1	2×2	-	256	-	-	-	C4
Convolution C5	3×3	1×1	256	Yes	GELU	circular (1)	M1
Convolution C6	3×3	1×1	512	Yes	GELU	circular (1)	C5
MaxPool M2	2×2	-	512	-	-	-	C6
AvgPool A1	2×2	-	512	-	GELU	-	M2
Transposed Conv T1	2×2	2×2	512	-	-	-	A1
GroupNorm G1	8×8	-	512	-	ReLU	-	T1
Input I2 (t)	-	-	1	-	-	-	-
Linear L1	-	-	512	No	GELU	-	I2
Linear L2	-	-	512	No	GELU	-	L1
Linear L3	-	-	512	No	GELU	-	I2
Linear L4	-	-	512	No	GELU	-	L3
Transposed Conv T2	2×2	2×2	256	No	-	-	G1 + L2
Convolution C7	3×3	1×1	256	No	GELU	circular (1)	T2 + C6
Convolution C8	3×3	1×1	256	No	GELU	circular (1)	C7 + C6
MaxPool M3	2×2	-	256	-	-	-	C8
Transposed Conv T3	3×3	1×1	256	No	-	-	M3 + L4
Convolution C9	3×3	1×1	256	No	GELU	None	T3 + C4
Convolution C10	3×3	1×1	256	No	GELU	None	C9
MaxPool M4	2×2	-	256	-	-	-	C10
Convolution C11	3×3	1×1	256	No	LeakyReLU	circular (1)	M4 + C1
GroupNorm G2	8×8	-	256	-	ReLU	-	C11
Convolution C12	3×3	1×1	1	No	LeakyReLU	circular (1)	G2
Optimizer	Adam lr=1e-4						
Batch size	32						
Epochs	3000						
Number of time steps	400						
Noise schedule	$\beta_1 = 1e-4$, $\beta_2 = 0.02$						

Table 4. Dimensions and training hyperparameters of the DDPM
